# On Arsenic and Sulphate of Quina in Acute Rheumatism

**Published:** 1826-02

**Authors:** John Whiting

**Affiliations:** Physician to the Surrey Dispensary.


### Art. III.—*On Arsenic and Sulphate of Quina in Acute Rheumatism.* By John Whiting, M.D. Physician to the Surrey Dispensary.

It not unfrequently happens that valuable remedies lose their hold on the good opinion of medical men, and are thrown aside, for want of a correct knowledge of their mode of operation. Impressed by this consideration, I cannot but hail, as a happy omen for our science, the desire lately excited amongst us to investigate more fully the peculiar effects of our medicinal agents. Towards this object I have lately been directing my attention, and, as my inquiries have already led to results which appear to me of some moment, I am induced to request you to allow your Journal to be the channel of communicating them to the profession.

A residence of several years in the vicinity of a marshy district in Norfolk, afforded me abundant opportunities of studying the nature and treatment of intermitting and remitting fevers. Among other facts which came to my knowledge, I observed that the objection which is made to the administration of bark during the paroxysms of these fevers, does not apply to the use of the arsenical solution. Indeed, I found, not only that there was no increase of fever, nor any unpleasant symptoms produced, but that, by continuing the medicine without any reference to

---

the paroxysms, especially in remittents, I gained much time in effecting a cure.*

A striking resemblance appearing to exist between the remittents under my care and the fever which accompanies acute rheumatism, I was led to make trial of the solution of arsenic in the latter of these diseases; my hopes of its utility being much encouraged by the strong testimony of Dr. Haygarth in favour of the cinchona.

I need not inform the medical reader, that, although the fever and inflammation which are attendant on acute rheumatism can be often quickly suspended by blood-letting and the general antiphlogistic treatment, yet the peculiar character of this disease, and that which renders it most difficult of cure, is its liability to pass from joint to joint, accompanied by fresh accessions of fever, and the tendency to the return of the symptoms, even after the patient has apparently recovered. It was with a hope of obviating this difficulty that I resorted to the trial of arsenic ; and it gives me pleasure to be able to state that my hopes have not been disappointed. For this purpose, I adopted the method of administration which I had found effectual in remittent fevers,—viz. from six to ten drops of the liquor arsenicalis, taken every three or four hours, without any regard to either the fever or inflammation. Under this treatment I discovered that, in many cases, none of the symptoms returned after the existing paroxysms had subsided; and that, in other instances, the future attacks became gradually weaker, and the patients were speedily cured.

Having thus satisfied myself that arsenic possesses the power of arresting the course of acute rheumatism, and knowing the close analogy which exists between the action of this medicine and the cinchona, I was induced to make trial of the sulphate of quinine; hoping, at the same time, that this beautiful preparation might also be found free from those objections which attach to the bark, when administered in substance during the existence of fever. The dose I gave was two grains, repeated every two or three hours.

After I had strictly watched its effects, I became convinced that this medicine, as well as arsenic, may be given with perfect safety both in remittent fever and acute rheumatism, even while febrile action is present; and that, in these diseases, it possesses the same property of wholly arresting, or greatly mitigating, the future paroxysms.

---

* I cannot help considering as groundless the fear which is entertained by many medical men of the effects of arsenic, when taken in the doses described in this letter; for I do not recollect, among the numerous cases which I have treated, any unpleasant occurrences arising from its administration.

I wish it, however, to be distinctly understood, that I have not discovered, in the operation of these remedies, any power of removing febrile or inflammatory action, when it has commenced. I attribute all the good which has resulted from their employment to some change produced in the system, by which, after a remission has taken place, a return of the symptoms is prevented. That they possess this valuable property in the cure of intermittents and remittents, has long been well known: that they possess the same power when employed in acute rheumatism, is the principal fact which I wish to make known by this communication.

From the foregoing observations it may be discovered, that the treatment suggested by them will not, in any degree, interfere with the antiphlogistic means which are usually employed to subdue existing fever or inflammation: indeed, the two measures may be most advantageously combined; for, while the one is calculated to cut short the paroxysm, the other either wholly prevents a return, or so mitigates future attacks as greatly to expedite a cure.

During a few months of the present year, while my attention has been directed to this subject, remittent fever and acute rheumatism have been prevalent in Southwark; and it appeared to me a duty to make known to some of my medical neighbours the substance of the preceding remarks, that any opportunity which should occur to them of aiding me in my researches might not be lost. By this means I have been favoured with cases corroborating my views; and it seems now to devolve upon me to make my opinions more public, in order that they may pass the ordeal of a more general investigation.

Before I conclude, I cannot refrain from observing, that I anticipate a great improvement in our treatment of many other diseases, by following up the indication which these medicines appear adapted to fulfil,—viz. the prevention of a periodic recurrence of symptoms. In gout, which in its character so much resembles acute rheumatism, I believe they may be administered with very great advantage. My friend, Mr. Greenwood, has lately had an opportunity of trying the effects of the sulphate of quinine in two cases of this disease, and he has informed me that in each of them it effectually prevented the recurrence of the paroxysms of pain, &c.

Hemicrania, tic douloureux, and many periodical diseases, are known occasionally to yield to the employment of arsenic and the quinine; and I do not think it unreasonable to indulge a hope that they may prove useful auxiliaries even in continued fevers, as remissions are frequently observed to take place, either spontaneously or from the effect of treatment. This subject is now occupying my attention; and the result of my inquiries, if it should appear to me worthy of notice, I shall have pleasure in communicating to the profession.

In the mean time, I am induced to request the attention of my medical brethren to the effect of the quinine and arsenic administered, in the way described in this letter, to patients labouring under acute rheumatism. Should any of your readers be led to give them a trial, it will afford me great satisfaction to learn the result, either through the medium of the public Journals or by private communication.

*Rodney Buildings, New Kent-road;*
*October* 1825.

